# Backbone-Minimised Nanoplasmid DNA Systems Enable High-Titre AAV Production in Suspension HEK293 Platforms

**DOI:** 10.3390/pharmaceutics18070814

**Published:** 2026-06-30

**Authors:** Lewis Hall, Michael J. Fiore, Joel Abramovich, Claire Kerridge, Ahad A. Rahim, Qasim A. Rafiq, Giulia Massaro

**Affiliations:** 1 Department of Biochemical Engineering, University College London, London WC1E 6BT, UK; 2 School of Pharmacy, University College London, London WC1N 1AX, UK; 3 Biotherapeutics and Advanced Therapies, Science and Research Group, Medicines and Healthcare Products Regulatory Agency, Potters Bar EN6 3GQ, UK

**Keywords:** AAV, Nanoplasmid^TM^, suspension culture, vector manufacturing, bioprocessing

## Abstract

**Background:** Scalability and cost remain major manufacturing barriers limiting broad patient access to adeno-associated virus (AAV) gene therapies. While capsid engineering has advanced vector biology, comparatively fewer innovations have addressed fundamental upstream productivity constraints. Transient triple-plasmid transfection is still the dominant AAV production platform and relies on large bacterial backbone plasmids that impose DNA burden and contribute significantly to the cost-of-goods. **Methods:** In this study, we evaluated a compact Nanoplasmid^TM^ DNA system (Aldevron) as a structural redesign of the transfection substrate to enhance upstream productivity. Conventional pUC-based triple-plasmid systems were compared to fully substituted Nanoplasmid^TM^ equivalents across suspension HEK293 production platforms optimised via response surface Design of Experiments. Hybrid plasmid configurations were also constructed to assess component-level contributions. **Results:** Complete substitution with Nanoplasmid^TM^ resulted in up to a 10-fold increase in vector genome titre relative to conventional plasmids under matched conditions. Hybrid systems failed to recapitulate this improvement, demonstrating that full-system backbone minimisation is required to ensure high yield. Productivity gains were preserved across transfection reagents and suspension media. Nanoplasmid^TM^ deployment represents a scalable, capsid-independent upstream intensification approach that improves yield without altering capsid biology. **Conclusions:** Integration of this approach within a design-for-manufacturability framework offers a practical route to reducing bacterial plasmid elements increasing safety, enhancing process robustness, and improving economic feasibility of AAV therapeutics.

## 1. Introduction

Recombinant adeno-associated virus (AAV) vectors have become the leading platform for in vivo gene delivery [1]. Approved therapies such as Luxturna, Zolgensma, and Hemgenix demonstrate the transformative potential of AAV-mediated gene transfer across ophthalmology [2], neuromuscular disease [3], and hematology [4]. Despite these clinical successes, the economic and logistical barriers associated with AAV manufacturing remain substantial. High therapeutic doses, often exceeding 10^14^ vector genomes (vg) per patient, combined with modest upstream productivity contribute to treatment costs measured in millions of dollars per dose [5,6]. Manufacturing scalability and cost remain major constraints limiting global patient access.

Transient triple-plasmid transfection in HEK293-derived cells remains the predominant manufacturing platform for clinical and commercial AAV production [7]; yet, this approach requires large quantities of plasmid DNA and exhibits modest volumetric productivity. This system relies on three plasmids encoding the ITR-flanked gene of interest (GOI) cassette, *Rep/Cap* genes, and adenoviral helper functions. Although flexible and widely adopted, this architecture carries inherent inefficiencies. Conventional plasmids contain large bacterial backbone regions, including antibiotic resistance genes and high-copy origins of replication, that are required for bacterial amplification but serve no function during mammalian transfection. These backbone elements increase plasmid size, elevate required DNA mass input, and may influence intracellular trafficking and transcriptional balance [8].

Residual plasmid DNA and plasmid recombination are safety concerns in recombinant AAV manufacturing. During transient transfection, fragments of plasmid DNA derived from transfer, *Rep/Cap*, or helper plasmids can be inadvertently encapsidated or co-purified with vector particles, resulting in residual DNA impurities in the final product. Although these sequences typically represent a small fraction of total nucleic acid content, they must be carefully characterised because they could theoretically lead to unintended gene expression, immunogenic responses, or other biological effects in treated patients [9]. Recent clinical observations have highlighted the potential relevance of contaminating plasmid sequences in vivo. For example, a recent study reported complex recombinant DNA structures in liver tissue following AAV gene therapy and suggested that manufacturing plasmid sequences might contribute to amplification or rearrangement of vector genomes within cells [10]. In addition, recombination events between plasmids during vector production can generate replication-competent AAV (rcAAV) through homologous or non-homologous recombination, representing a potential safety concern if not properly controlled [11]. Consequently, AAV manufacturing processes need to incorporate plasmid design strategies, purification workflows, and analytical testing to minimise residual plasmid DNA and detect potential recombination products. From a regulatory perspective, residual plasmid DNA and recombination-derived species are critical quality attributes that must be controlled during AAV vector manufacturing. Regulatory agencies require characterisation and quantification of residual host cell DNA, plasmid DNA, and rcAAV as part of Chemistry, Manufacturing and Controls packages for gene therapy products; however, products are reviewed on a case-by-case basis so there remains no harmonised parameters. Residual DNA is typically controlled through validated purification processes and sensitive quantitative assays such as qPCR or digital PCR. In addition, rcAAV assays are implemented to detect recombination events that may reconstitute functional *rep* and *cap* sequences during production. The presence of complex recombinant DNA species in tissues following AAV therapy further highlight the importance of minimising plasmid-derived contaminants and homologous recombination substrates during manufacturing to decrease the burden of plasmid-derived impurities in final vector preparations [5].

Process intensification efforts have traditionally focused on optimising transfection chemistry, media formulation, or bioreactor configuration [7,12]. However, relatively limited attention has been directed toward redesigning the plasmid material itself. Nanoplasmid^TM^ (Aldevron) are compact, antibiotic-free DNA constructs designed to minimise bacterial backbone sequences while retaining essential mammalian expression elements [13]. Reduced plasmid size has been associated with improved transfection efficiency and expression kinetics in multiple mammalian systems [14].

While the Nanoplasmids^TM^ technology has been made commercially available by Aldevron in 2022, a systematic analysis of AAV manufacturing performance in suspension cells has not been performed before. We hypothesised that full-system replacement of conventional triple-transfection plasmids with Nanoplasmid^TM^ could enhance AAV productivity independent of capsid identity by reducing DNA burden and improving intracellular processing efficiency. Complete conventional and complete Nanoplasmid^TM^ systems were compared under identical conditions to establish the baseline advantage of compact backbones. Mass-based versus molar-based plasmid ratioing was tested across the time course of production to determine whether equimolar delivery is required to fully realise Nanoplasmid^TM^ benefits. Different transfecting reagents were also compared on both DNA platforms to ensure that gains were not reagent-specific. Finally, hybrid mixtures that swap individual Nanoplasmid^TM^ components into an otherwise conventional system were used to investigate whether benefits are additive or require a fully coherent three-plasmid Nanoplasmid^TM^ set. Helper:*Rep/Cap*:transgene ratios were tuned in molar terms to identify whether the optimal stoichiometry shifts between two different P5 architectures. A clinically relevant transgene was selected based on prior work developing an AAV vector delivering the human *GBA1* gene [15,16] for the treatment of a severe and rare neurodegenerative disorder, Gaucher Disease, to enable evaluation within a clinically relevant therapeutic context. By establishing a scalable, high-yield manufacturing process while maintaining the previously established vector qualities, this work supports the future clinical development and broader accessibility of gene therapy approaches for genetic disorders like Gaucher Disease. In addition, the use of suspension-adapted HEK293 cells in this study provides a manufacturing model that reflects current industrial AAV production strategies, strengthening the translational relevance of our findings and aligning with established upstream process development workflows for gene therapy manufacturing.

## 2. Materials and Methods

*Cell Lines and Culture Systems*

Suspension HEK293F cells (Thermo Fisher Scientific, Waltham, MA, USA, catalogue no. R78005) were maintained in HyClone™ SFM4HEK293 (Peak) serum-free medium (Cytiva, Wilmington, DE, USA, catalogue no. SH30521.02) in 125 mL polycarbonate Erlenmeyer flasks (Corning, New York, NY, USA, catalogue no. 431143) at a working volume of 30 mL. Cells were cultured at 37 °C with 8% CO_2_ and agitated at 145 rpm. Viable cell density and viability were monitored using the NucleoCounter NC-200 automated cell counter (ChemoMetec, Allerod, Denmark). For adherent culture experiments, HEK293T cells were seeded at 50,000 cells/cm^2^ in DMEM supplemented with 10% *v*/*v* foetal bovine serum (FBS) and cultured at 37 °C with 5% CO_2_. Cultures were gently resuspended by swirling, then a 0.5 mL sample was removed daily for counting on a NucleoCounter NC-200 using the manufacturer’s protocol, recording viable cell density, viability, mean diameter, and percent aggregates. Cultures were passaged every 2–3 days, or whenever viable cell density approached 2.5–3.0 × 10^6^ cells/mL, to maintain cells within the recommended range. Cultures were harvested at 72 h post-transfection unless otherwise specified. At the point of harvest, the culture was collected and AAV was released using AAVMAX lysis buffer (Thermo Fisher Scientific, catalogue no. A35684) according to the manufacturer’s lysis workflow.

*Plasmid Systems*

Two plasmid systems were evaluated. The conventional system consisted of pUC-based plasmids encoding helper, *Rep/Cap*, and ITR-flanked transgene components. These plasmids contained standard bacterial origins of replication and Ampicillin antibiotic resistance markers. The Nanoplasmid^TM^ system was designed by the authors and manufactured by Aldevron. It consisted of size-minimised, antibiotic-free constructs in which bacterial backbone elements were substantially reduced. Briefly, Nanoplasmids^TM^ are produced by Aldevron using a specialised bacterial amplification system that replaces conventional antibiotic-resistance selection with an RNA-based sucrose selectable marker. Following transformation into a proprietary *E. coli* production strain, the Nanoplasmid^TM^ replicates at high copy number while maintaining a minimal bacterial backbone. The resulting construct contains only the expression cassette, a minimal origin of replication, and the selection marker, reducing non-essential bacterial DNA by up to 70–90% compared with conventional pUC-derived plasmids. The expression cassettes of the original pUC plasmids were retrofitted into the Nanoplasmid^TM^ Aldevron system. Two *Rep/Cap* variants were tested: a standard truncated P5 promoter configuration and a direct-retrofit variant incorporating a P5-TATA modification. To interrogate system-wide effects, hybrid configurations were constructed in which single plasmid components were substituted between conventional and Nanoplasmid^TM^ systems.

*Suspension Transfection Optimisation and Downstream Capture*

For suspension culture experiments, HEK293F cells were seeded at 1 × 10^6^ cells/mL and transfected 24 h post-seeding. Plasmid complexes were prepared in Opti-MEM I Reduced Serum Medium (Thermo Fisher Scientific, Waltham, MA, USA, catalogue no. 31985062) at a volume corresponding to 10% of the culture volume and incubated for 20 min at room temperature prior to dropwise addition to the culture. PEIpro (Polyplus, Illkirch, France, catalogue no. 101000007) was used as the transfection reagent for all suspension experiments. A four-factor response surface Design of Experiments (DoE) model was employed to optimise transfection parameters (JMP Pro 18). Factors included total DNA amount per 10^6^ cells, PEI:DNA mass ratio, complexation time, and complexation volume expressed as a percentage of culture volume. Thirty-three runs including replicated centre points were executed. Key growth parameters, including Viable Cell Density (VCD), cell diameter, specific growth rate calculated as the rate of increase in cell population per unit of existing population over time (μ=ln(Nt−N0)/∆t), and population doubling time, were monitored. Vector genome titre (vg/mL) was quantified via qPCR targeting the transgene sequence following DNase treatment. “Baseline conditions” refers to the pre-optimisation reference transfection used before the DoE-informed parameters were applied: suspension HEK293 cells transfected in HyClone™ Peak medium without supplementation, using 1 µg DNA per 10^6^ cells with PEIpro at a 2:1 PEI:DNA mass ratio, complexes incubated for 15 min at room temperature, and an addition volume of 5% of the culture volume.

*Adherent Transfection for Plasmid System Comparison*

For plasmid system comparison experiments, AAV9 was produced in adherent HEK293T cells seeded at 50,000 cells/cm^2^ and transfected 24 h post-seeding using PEIpro (Polyplus, catalogue no. 101000007)-mediated triple transfection, except for transfection reagent comparison experiments in which both PEIpro and linear PEI MAX (MW 40,000; Polysciences, Warrington, PA, USA, catalogue no. 24765) were evaluated. Complexes were prepared in Opti-MEM I Reduced Serum Medium (Thermo Fisher Scientific, catalogue no. 31985062) at a volume corresponding to 10% of the culture volume and incubated for 20 min at room temperature. Plasmids were used at a total DNA input of 1 µg per 10^6^ cells at a PEI:DNA mass ratio of 2:1. For plasmid system and stoichiometry experiments, plasmids were combined at a 3:1:1 ratio (helper:*Rep/Cap*:transgene) prepared either by mass or molar equivalence depending on the experimental condition. The pAAV.hSYN.hGBA1.WPRE transgene plasmid was used for all conventional system experiments; pAAV.hSYN.hGBA1.WPRE-NP was used for all Nanoplasmid^TM^ system experiments. To examine the effect of plasmid backbone format, eight configurations were evaluated: an all-Nanoplasmid^TM^ system, an all-conventional system, and six hybrid systems in which a single component (helper, *Rep/Cap*, or transgene) from one platform was substituted into the other. Three biological replicates were used per condition.

*Vector Genome Titre Quantification*

Vector genome titre was quantified by quantitative polymerase chain reaction (qPCR) using a StepOnePlus Real-Time PCR System (Thermo Fisher Scientific, Cheshire, UK). Benzonase nuclease treatment (150 U/mL; Merck, Darmstadt, Germany, catalogue no. E1014) was performed as part of the AAVMAX lysis protocol prior to qPCR analysis. Absolute quantification was performed using ITR-targeting primers with SsoAdvanced Universal SYBR Green Supermix (Bio-Rad, Hercules, CA, USA) against a linearised plasmid standard curve. Specificity was confirmed by melt curve analysis. Results are expressed as vector genomes per mL (vg/mL) or per cm^2^ (vg/cm^2^) as stated.

*Full: empty capsid quantification*

An amount of 5 μL of sample was loaded into the mass photometer (Samux, Refeyn, Oxford, UK) and reading parameters were set according to the estimated concentration. Packaged genome mass was calculated by subtracting empty capsid mass from full capsid mass.

*Statistical Analysis*

Statistical analyses were performed using one-way or two-way ANOVA with Tukey’s multiple comparison test, implemented in GraphPad Prism 10 (GraphPad Software, San Diego, CA, USA). Response-surface DoE modelling was performed using JMP Pro 18 (SAS Institute, Cary, NC, USA). Significance was defined as *p* < 0.05.

*Generative AI tool*

The graphical abstract was prepared using Google Gemini.

## 3. Results

### 3.1. Suspension Transfection Optimisation Established a High-Performance Baseline

**Figure 1:**
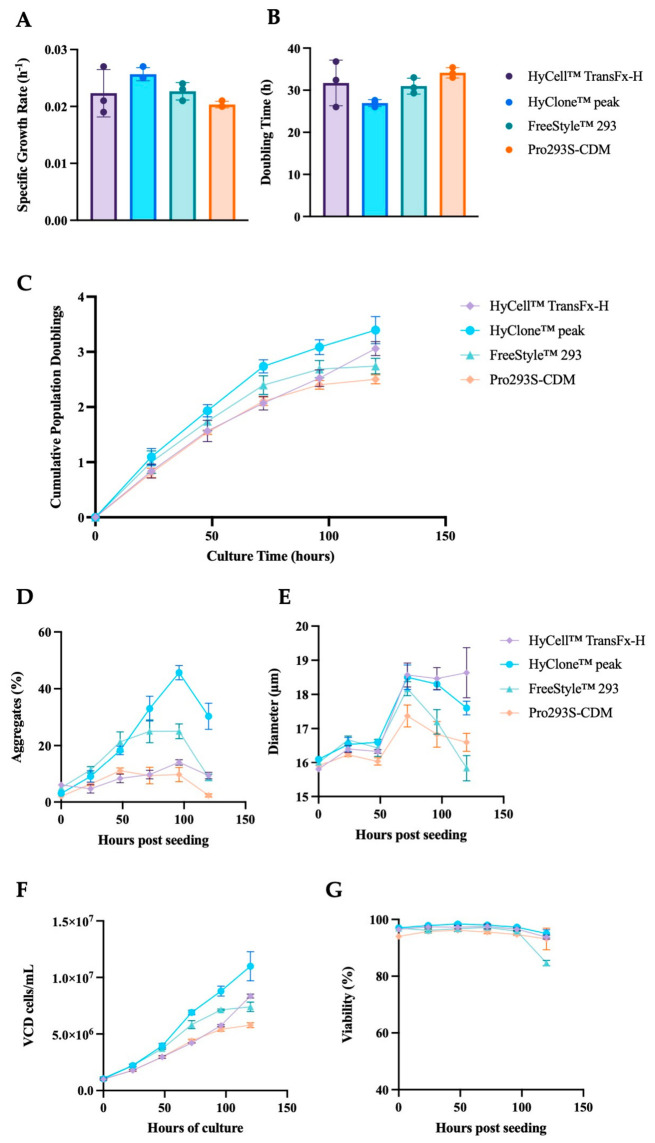
Optimisation of suspension culture, growth kinetics, and population dynamics across four serum-free media. (**A**) Specific growth rate (μ) over 0–72 h. (**B**) Doubling time. (**C**) Cumulative population doublings plotted over culture time, increasing to ~2.3–2.7 by 72 h with the same performance ranking. (**D**) Aggregate fraction. (**E**) Cell diameter. (**F**) Viable cell density over 0–120 h. (**G**) Viability. Points show condition means with error bars where visible (±SD).

Suspension culture development was undertaken to establish a scalable upstream platform suitable for evaluating plasmid system performance during AAV production. HEK293F cells were cultured in multiple commercially available serum-free media formulations (HyCell™ TransFx-H, HyClone™ Peak, FreeStyle™ 293, and Pro293-CDM) to identify conditions supporting robust growth and high-density culture performance (Figure 1A–C). Comparative growth analysis demonstrated clear differences in viable cell density, doubling time, and long-term viability between formulations (Figure 1D–G). Overall, HyClone™ Peak medium performed better than the other alternatives and supported stable suspension growth with minimal aggregation and reproducible expansion to high cell densities.

**Figure 2:**
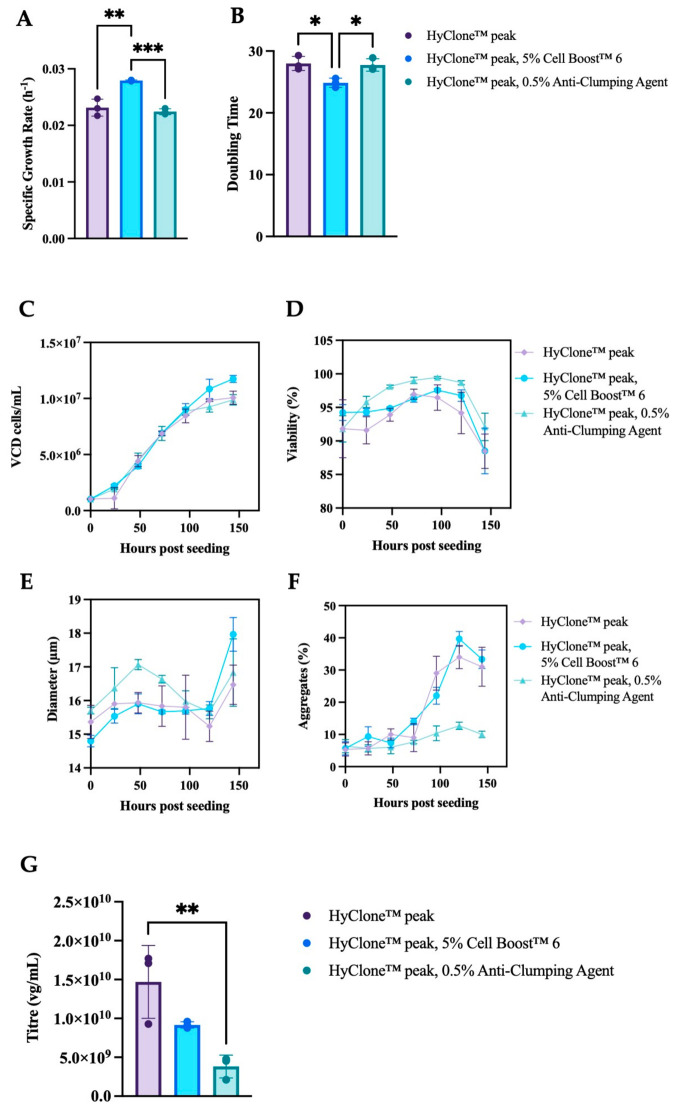
HyClone™ Peak Media Optimisation. (**A**) Specific growth rate (μ) over the exponential phase. (**B**) Doubling time. Points denote individual measurements; horizontal bars indicate means. Statistical significance is annotated in the plot (one-way ANOVA with Tukey’s test: * *p* ≤ 0.05; ** *p* ≤ 0.01; *** *p* ≤ 0.001). (**C**) Viable cell density (VCD) over 0–144 h. (**D**) Viability. (**E**) Cell diameter. (**F**) Aggregate fraction. Points show means with error bars (±SD). (**G**) Effect of HyClone™ Peak supplements on AAV titre. qPCR-measured vector genome titre for cultures in HyClone™ Peak (control), HyClone™ Peak + 5% Cell Boost™ 6, and HyClone™ Peak + 0.5% Anti-Clumping Agent (ACA). Statistical annotation on the plot indicates a significant decrease versus control (** *p* ≤ 0.01; one-way ANOVA with Tukey’s test). Bars show mean ± SD (*n* = 3).

Building on this foundation, we investigated the potential for targeted supplementation to enhance culture performance, evaluating both Cell Boost™ 6 (CB6) as a nutrient-support lever, aimed at sustaining metabolism at higher cell densities and preserving late-run viability, and an anti-clumping agent (ACA) as a mechanistic probe for morphological control, rather than as a production additive. The rationale for including ACA was based on its known ability to mitigate aggregation in suspension cultures, although its surfactant-based chemistry is documented to interfere with transient PEI-mediated transfection, particularly during the critical window of polyplex uptake, thereby typically precluding its use in production contexts. Accordingly, we designed comparative experiments examining three conditions: HyClone™ Peak alone, Peak supplemented with 5% CB6, and Peak supplemented with 0.5% ACA monitoring cell growth (Figure 2A,B), viable cell density, viability, cell diameter, and aggregate fraction over a 144-h culture period (Figure 2C–F). The addition of 5% CB6 resulted in measurable improvements in growth kinetics, reducing the population doubling time from approximately 27.5 h in the control to around 25 h and increasing the specific growth rate from 0.023 h^−1^ to 0.028 h^−1^. In contrast, the ACA-supplemented cultures exhibited intermediate performance, with a doubling time of approximately 27 h and a specific growth rate of 0.025 h^−1^, indicating that while ACA can modestly influence proliferation, its primary functional benefit is likely in morphological control rather than acceleration of growth. Viable cell densities over the 144-h period were largely similar across conditions, although CB6-supplemented cultures demonstrated a modest late-stage advantage, with viability remaining near or above 90% at 144 h; whereas, control and ACA cultures declined toward the mid-80s after approximately 100 h. Cell diameters increased from 15 to 16 μm early in the run to 17–18 μm mid-run and remained stable across conditions, demonstrating that neither CB6 nor ACA introduced detectable morphological perturbations. Aggregation behaviour, however, displayed a pronounced dependence on supplementation: ACA effectively minimised aggregate formation throughout the culture, whereas CB6 tended to support slightly higher aggregation in late-stage cultures, with the control intermediate between these extremes, consistent with the mechanistic role of ACA in reducing cell–cell interactions.

An assessment of AAV vector genome titres (Figure 2G) revealed a nuanced interplay between growth and transfection efficiency: cultures in Peak alone achieved the highest titres of 1.47 × 10^10^ vg/mL, CB6 supplementation produced a slightly lower, though not statistically significant, titre of 9.0 × 10^9^ vg/mL, and ACA reduced titres substantially to 4.5 × 10^9^ vg/mL (*p* < 0.01). This reduction aligns with the known interference of surfactant-based additives with PEI-DNA polyplex formation, likely due to steric hindrance or altered electrostatic interactions at the cell membrane, impairing plasmid delivery and subsequent co-expression of *Rep/Cap* and helper functions critical for AAV assembly. Despite CB6’s ability to improve late-stage viability and marginally increase peak cell densities, its lack of effect on titres suggests that cell proliferation was not the rate-limiting step under baseline transfection conditions and highlights the possibility that components of CB6 may have unintended interactions with polyplex stability or cellular uptake. In addition, the higher cell density in the CB6 samples can cause contact inhibition resulting in poor uptake of nucleic acids and decreased gene expression. The observed titre range of approximately 10^12^ vg/L (10^9^ vg/mL) is consistent with literature-reported yields for HEK293 suspension cultures under serum-free conditions, where optimised systems typically achieve 10^12^–10^14^ vg/L, thereby validating the experimental baseline.

### 3.2. DoE Experimental Rationale and Design

Following medium selection, a multivariate optimisation strategy was applied to define optimal transient transfection parameters in suspension culture using conventional DNA plasmid material. Using a response surface Design of Experiments, interactions between total DNA input, PEI:DNA ratio, complexation time, and complexation volume were systematically evaluated. This approach identified a defined operational window that significantly improved AAV vector genome titres compared with baseline transfection conditions while maintaining high cell viability.

**Figure 3:**
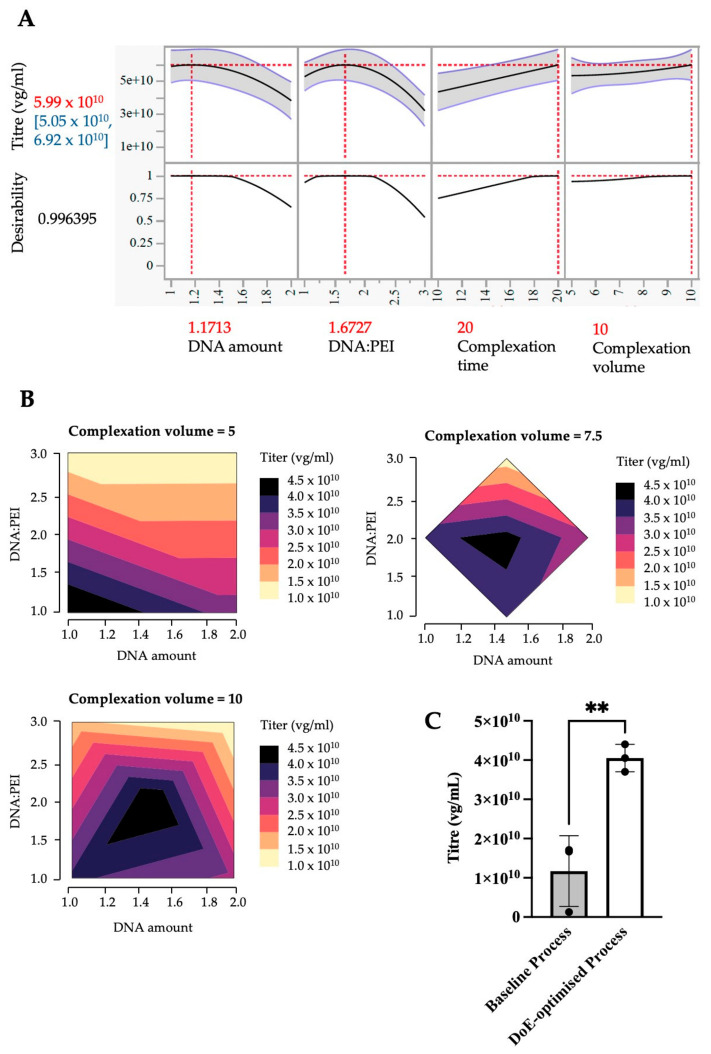
DoE Experimental Rationale and Design. (**A**) Prediction profiler and desirability for response-surface optimisation of rAAV titre. JMP Pro 18 prediction profiler showing modelled effects of four transfection factors on vector genome titre (vg/mL). Top panels plot the fitted response with 95% confidence bands across the studied ranges. Bottom panels show the individual desirability function for titre. Vertical red dashed lines mark the settings that maximise desirability. (**B**) Contour plots of predicted rAAV titre as a function of DNA amount and PEI:DNA ratio at fixed complexation volumes. The contour lines represent regions of equal predicted response, with warmer colours indicating higher vector yields and cooler colours indicating lower yields. The region enclosed by the highest-value contours identifies the predicted optimum operating space, where a balance between the two parameters maximises vector production. Conversely, movement away from this region in any direction results in progressively lower titres, indicating that excessive or insufficient values of either factor are detrimental to process performance. Response-surface model contours (quadratic fit; *n* = 33 runs) showing the predicted vector genome titre across DNA amount (μg per 10^6^ cells, x-axis) and PEI:DNA mass ratio (y-axis) at three complexation volumes: 5%, 7.5%, and 10% *v*/*v*. (**C**) Validation of response-surface optimisation for suspension transfection. Vector genome titre from baseline conditions versus DoE-informed settings derived from the prediction profiler/desirability maximum (optimised set of conditions (DNA 1.2 μg per 10^6^ cells, PEI:DNA 1.5:1, complexation 20 min, complexation volume 10% *v*/*v*). Bars show mean ± SD (*n* = 3); statistical annotation indicates a significant difference (** *p* ≤ 0.01).

Given the critical influence of polyplex formation on transient PEI-mediated transfection, a multivariate, response-surface DoE was employed, encompassing four factors: DNA amount per 10^6^ cells (1.0, 1.5, 2.0 μg), PEI:DNA mass ratio (1:1, 2:1, 3:1), complexation time (10, 15, 20 min), and complexation volume as a percentage of culture volume (5%, 7.5%, 10%). A total of 33 runs (27 factorial points plus six replicated centre points) were conducted in 2 mL 6-well suspension plates seeded at 1 × 10^6^ cells/mL in HyClone™ Peak, with vector genome titres measured at 72 h post-transfection by qPCR serving as the primary readout (Figure 3A). The three discrete levels tested (1:1, 2:1, 3:1), together with the other factor levels and the replicated centre points across the 33-run design, were used to fit a continuous quadratic model for vector genome titre. The JMP Pro 18 prediction profiler then identified the PEI:DNA setting that maximises titre/desirability across this continuous factor space, which fell at a modelled optimum of approximately 1.672:1. The resulting quadratic model was statistically robust (R^2^ = 0.874; adjusted R^2^ = 0.845; *p* < 0.0001), with DNA:PEI ratio emerging as the dominant determinant of titre, supported by highly significant linear (*p* < 0.0001) and quadratic (*p* = 0.00028) terms. DNA amount exerted a significant, albeit less pronounced, effect (*p* = 0.012), with a marginal quadratic trend (*p* = 0.053) suggesting diminishing returns above 2 μg/10^6^ cells, while complexation time contributed significantly (*p* = 0.027), favouring intermediate incubation periods to balance adequate polyplex formation without promoting over-condensation. Notably, a significant interaction between DNA amount and complexation time (*p* = 0.0073) underscored the sensitivity of polyplex kinetics to plasmid loading, highlighting the necessity for precise coordination of complexation parameters.

Complexation volume influenced titres in a manner dependent on both DNA amount and PEI ratio, with larger volumes (7.5–10%) demonstrating a defined optimum centred around 1.5 μg DNA per 10^6^ cells and a DNA:PEI ratio of 2–3:1, predicting titres exceeding 5 × 10^10^ vg/mL (Figure 3B). Contour surface visualisations presented a response illustrating the combined effects of the two investigated variables (DNA concentration versus PEI:DNA ratio) on AAV titre and confirmed these factor interactions and provided actionable insights for parameter selection. Model predictions were validated experimentally using 1.2 μg DNA/10^6^ cells, 1.6:1 PEI:DNA ratio, 20-min complexation, and 10% complexation volume, yielding 4.05 × 10^10^ vg/mL, a 2.2-fold improvement over the baseline of 1.47 × 10^10^ vg/mL (Figure 3C).

These results corroborate prior studies demonstrating that transient transfection efficiency and rAAV productivity are tightly coupled to narrow polyplex formation optima, with deviations leading to marked declines in titre [17]. Collectively, our findings establish a two-tiered strategy for process optimisation: (i) selection of a robust serum-free base medium, such as HyClone™ Peak, which provides high baseline growth and viability without introducing confounding interactions with polyplex uptake, and (ii) precise tuning of PEI-mediated transfection parameters, including DNA amount, PEI:DNA ratio, complexation time, and volume, to maximise vector genome yield. The optimised protocol proved robust and readily reproducible across replicate cultures, demonstrating that efficient transient transfection could be achieved in serum-free suspension conditions without complex handling procedures.

### 3.3. Nanoplasmid^TM^ Substitution Increases AAV Vector Genome Titre

**Figure 4:**
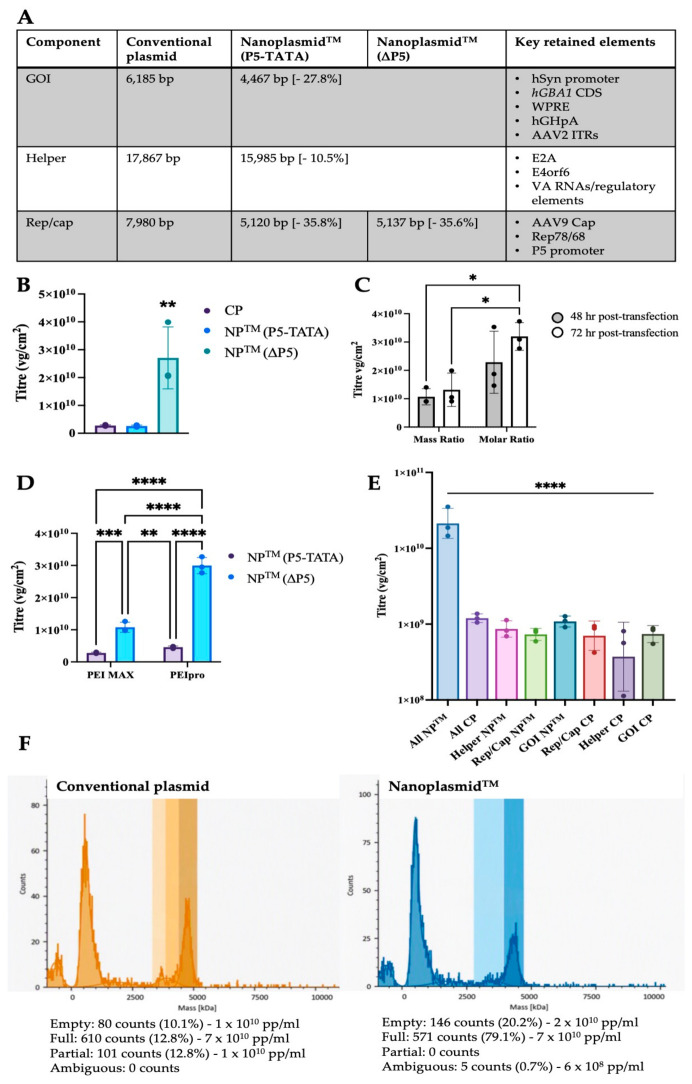
Comparative Evaluation of Conventional versus Nanoplasmid^TM^ Systems. (**A**) Conventional plasmids and Nanoplasmid^TM^ counterparts used for AAV9 triple transfection. Sizes are in base pairs (bp). Reductions are calculated versus the conventional construct. The direct-retrofit Nanoplasmid^TM^ differs from the standard Nanoplasmid^TM^ only for the *Rep/Cap* vector (enhanced P5 with TATA addition). (**B**) Vector genome titre obtained with the conventional pUC-based triple-plasmid system (CP), the Nanoplasmid^TM^ (NP^TM^) (direct retrofit set (P5-TATA), and the Nanoplasmid^TM^ (standard) set (truncated P5). Bars show mean ± SD (*n* = 3). Statistics: one-way ANOVA followed by Tukey’s test (** *p* ≤ 0.01). (**C**) Effect of mass- versus molar-based plasmid ratios on AAV9 titre at 48 h and 72 h. Vector genome titre for mass-ratio and molar-ratio transfections using the Nanoplasmid^TM^ system (3:1:1). Bars show mean ± SD (*n* = 3). Statistics: one-way ANOVA with Tukey’s multiple comparisons across the four groups (* *p* ≤ 0.05). (**D**) AAV9 titres for PEI MAX versus PEIpro across conventional and Nanoplasmid^TM^ plasmid systems. Vector genome titre for the conventional pUC-based system (CP) and the Nanoplasmid^TM^ (NP^TM^ ) system transfected with either PEI MAX or PEIpro. Bars show group means ± SD (*n* = 3). Statistics: two-way ANOVA followed by one-way ANOVA with Tukey’s multiple comparisons (** *p* ≤ 0.01, *** *p* ≤ 0.001, ****, *p* < 0.0001). (**E**) Impact of hybrid plasmid/Nanoplasmid^TM^ configurations on AAV9 titre. Vector genome titre for eight configurations: all-Nanoplasmid^TM^ (NP^TM^), all-conventional (CP), and six hybrids in which a single component (helper, *Rep/Cap*, or GOI) from one platform was substituted into the other. Bars show mean ± SD (*n* = 3). Statistics: one-way ANOVA with Tukey’s multiple comparisons; the all-Nanoplasmid^TM^ group differed significantly from every other configuration (****, *p* < 0.0001), while the all-conventional and all hybrid groups did not differ from one another (ns). (**F**) Mass photometry analysis showing overlay of particle mass distribution, with estimation of full and empty capsids.

To evaluate the impact of plasmid architecture on rAAV9 production, a series of Nanoplasmid^TM^ variants were engineered and systematically compared to conventional pUC-based plasmids in the context of a triple-transfection system. Each conventional plasmid was retrofitted to a Nanoplasmid^TM^ counterpart (Figure 4A), preserving all essential functional elements while replacing the pUC-derived backbone with a compact Nanoplasmid^TM^ backbone of less than 500 bp, implementing RNA-OUT antibiotic-free selection and an R6K origin that restricts propagation to specialised hosts, thereby eliminating most antibiotic resistance markers and non-essential bacterial sequences. The transgene construct, pAAV.hSYN.hGBA1.WPRE-NP, was reduced from 6185 bp to 4467 bp, retaining the hSYN promoter, *GBA1* coding sequence, WPRE, and hGH polyadenylation signal, flanked by functional AAV2 ITRs. The helper plasmid, pHGTI SoP-NP, was reduced from 17,867 bp to 15,985 bp while maintaining E2A, E4orf6, VA RNAs, and associated regulatory elements. Two *Rep/Cap* Nanoplasmid^TM^ were designed: the standard Nanoplasmid^TM^ pDG9 SoP-NP (5137 bp) using a truncated P5 promoter, and the direct-retrofit pDG9 SoP-NP (5120 bp) incorporating a P5 promoter with a canonical TATA site to support *Rep* transcriptional initiation. At the system level, the total DNA content decreased from 32,032 bp in the conventional system to 25,589 bp for the standard Nanoplasmid^TM^ set and 25,572 bp for the direct-retrofit set, representing 20.1–20.2% reduction in DNA content. Direct comparison of rAAV9 production under identical transfection conditions (equal total DNA mass per cell with all polyplex- and process-related parameters held constant) revealed a clear advantage for the standard Nanoplasmid^TM^ design (Figure 4B), achieving mean titres of 2.71 × 10^10^ vg/cm^2^, compared to 2.77 × 10^9^ vg/cm^2^ for the conventional system and 2.59 × 10^9^ vg/cm^2^ for the direct-retrofit design, corresponding to approximately 9.8-fold and 10.5-fold improvements, respectively, with all other variables held constant, including total DNA mass, plasmid ratios, PEI:DNA ratio, complexation time and volume, addition volume, seeding density, medium, and harvest time.

Mechanistically, two primary features likely contribute to this performance gain: the minimised Nanoplasmid^TM^ backbone, which removes non-essential bacterial sequences and antibiotic markers, reducing cytotoxicity and silencing while improving transfection efficiency, and the truncated P5 promoter in the standard Nanoplasmid^TM^, which provides moderated Rep78/68 expression levels; this is critical given the sensitivity of rAAV replication to *Rep* expression, where overexpression inhibits, and finely tuned expression enhances, viral yield. The direct-retrofit P5-TATA Nanoplasmid^TM^, despite sharing the compact backbone, failed to outperform the conventional system, suggesting that the P5 architecture, rather than backbone size alone, is the primary determinant of replication efficiency and overall titre in this context.

Furthermore, optimisation of plasmid stoichiometry demonstrated that delivering plasmids on a molar rather than mass basis substantially enhances production, particularly with the Nanoplasmid^TM^ platform; switching from a mass-based 3:1:1 (helper:*rep/cap*:transgene) ratio to a molar-based 3:1:1 ratio restored equimolar molecular delivery, accounting for differences in plasmid size (15,985/5137/4467 bp), and resulted in ~2.1-fold titre increases at 48 h and ~2.4-fold increases at 72 h post-transfection (Figure 4C). This finding underscores the critical importance of precise stoichiometric balance in triple-transfection systems, as suboptimal delivery of helper plasmids relative to *Rep/Cap* and transgene constructs can limit co-transfection efficiency during the peak genome replication phase [18]. All other parameters influencing polyplex formation, including PEI:DNA ratio, complexation time, and volume, were kept constant, isolating the stoichiometry effect and confirming that the observed gains are attributable to molar-based plasmid ratios rather than variations in complexation conditions.

Comparative evaluation across transfection reagents (Figure 4D) further highlighted platform-dependent interactions: with a 3:1:1 molar ratio, PEI MAX yielded 2.77 × 10^9^ vg/cm^2^ for the conventional system versus 1.08 × 10^10^ vg/cm^2^ for the Nanoplasmid^TM^ platform (~3.9-fold gain); whereas, PEIpro produced 4.58 × 10^9^ vg/cm^2^ for the conventional platform and 3.00 × 10^10^ vg/cm^2^ for the Nanoplasmid^TM^ (~6.6-fold gain). Within-platform reagent effects were modest for the conventional system but substantial for Nanoplasmid^TM^, where PEIpro produced an additional ~2.8-fold increase compared to PEI MAX. These observations suggest that plasmid size and topology interact with polyplex physiochemistry, including size, surface charge, and unpacking efficiency, to influence intracellular delivery and co-expression of helper, *Rep/Cap*, and transgene constructs.

Taken together, the data establish a robust framework for leveraging Nanoplasmid^TM^ platforms in HEK293 suspension cultures: by combining backbone minimization, tailored *Rep* promoter design, precise molar stoichiometry, and an appropriate PEI reagent, rAAV production can be enhanced substantially, providing a scalable, reproducible, and mechanistically informed strategy for high-yield vector manufacture, consistent with reports in the literature on the influence of plasmid size, promoter architecture, and polyplex physiochemistry on transient transfection efficiency in mammalian expression systems. This integrated approach highlights the importance of evaluating both molecular design and delivery context to achieve maximal vector genome production and suggests that Nanoplasmid^TM^ platforms may represent a broadly applicable solution for improving rAAV yields across diverse capsid and transgene configurations.

### 3.4. Hybrid Configurations Demonstrate Requirement for Full-System Replacement

To systematically dissect the contribution of plasmid architecture to rAAV9 productivity, we compared eight configurations encompassing all-Nanoplasmid^TM^, all-conventional, and six hybrid sets in which a single component, either the helper, *Rep/Cap*, or transgene plasmid, was swapped between the Nanoplasmid^TM^ and conventional platforms while maintaining all other variables constant, including total DNA per well, PEI:DNA ratio, complexation time and volume, addition volume, seeding density, medium composition, and harvest time (Figure 4E). Strikingly, a clear binary pattern emerged: only the all- Nanoplasmid^TM^ configuration achieved high productivity, with mean titres of approximately 2.3 × 10^10^ vg/cm^2^; whereas, the all-conventional system and all six hybrid constructs clustered in a lower yield range around 10^9^ vg/cm^2^, revealing that partial incorporation of Nanoplasmid^TM^ elements was insufficient to confer intermediate gains. This all-or-nothing behaviour suggests that the mechanistic advantages conferred by Nanoplasmid^TM^ engineering are not independently additive at the single-component level but require coordinated co-optimisation across all three plasmids in the triple-transfection system. In the context of triple-plasmid rAAV production, efficient viral assembly relies on the fraction of cells that simultaneously co-receive and express all three essential components in appropriate stoichiometric proportions; vector genome yield scales directly with this triple-positive fraction and is inherently constrained by the least efficient component.

Mass photometry analysis was performed to characterise capsid packaging properties of Nanoplasmid^TM^ and conventional plasmid-derived AAV preparations (Figure 4F). AAV manufactured with Nanoplasmid^TM^ maintained high percentage of full capsids (79.1%) comparable to traditional plasmids-derived preparation (77.1%) at the same capsid concentration (7 × 10^10^ pp/mL), with a total full:empty ratio of 3.91 compared to 7.62 of plasmids-derived preparations, and showed a drastic reduction in partially full capsids (partial or ambiguous counts: 0.7% Nanoplasmid^TM^, 12.8% conventional plasmids). In addition, particle analysis revealed that the mass of the packaged genome in the AAV preparation manufactured with Nanoplasmid^TM^ was 4021 kDa, compared to 4117 kDa for conventional plasmids. When normalised to theoretical construct mass (2952 kDa for nanoplasmid; 4082 kDa for conventional), Nanoplasmid^TM^ demonstrated a packaging efficiency of 136.2% compared to 100.9% for conventional plasmids.

## 4. Discussion

In this study we optimised culturing conditions of suspension HEK293F cells using DoE, and evaluated the use of Nanoplasmid^TM^ technology to lower DNA burden and regulatory risks during viral vector manufacturing. We demonstrated that complete substitution of conventional pUC plasmids with Nanoplasmid^TM^ constructs under optimised growing conditions significantly increased AAV vector genome yield in suspension HEK293 cultures.

The failure of hybrid systems to achieve even partial enhancement indicates that the inclusion of a single conventional plasmid disrupts this delicate balance, effectively creating the weakest link that governs system output, such that overall titres revert to the baseline observed with entirely conventional constructs. From a molecular perspective, the underlying cause is likely rooted in plasmid size and backbone differences: Nanoplasmid^TM^ features compact backbones (<500 bp), RNA-OUT antibiotic-free selection, and R6K origins; whereas, conventional pUC-based vectors are larger and contain non-essential bacterial sequences and antibiotic resistance markers. When plasmids of differing size and topology are combined, especially under mass-based dosing [19], the resulting delivered molecule numbers per component are uneven, deviating from the intended stoichiometry [18] required for coordinated Rep-mediated genome replication, capsid expression, and genome packaging. For example, the helper plasmid is substantially larger than the transgene in conventional form and mixing it with a smaller Nanoplasmid^TM^ counterpart at the same mass leads to underrepresentation in molecular terms. This, in turn, limits the fraction of cells that successfully co-express all three plasmids as co-transfection is stochastic and plasmid length and composition influence intracellular delivery [20,21]. This effect is compounded by the sensitivity of transient PEI-mediated transfection to polyplex formation and unpacking kinetics: differences in plasmid length and backbone composition alter the condensation, charge distribution, and intracellular processing of PEI–DNA complexes [22,23], potentially affecting nuclear delivery and timing of expression [24] across components. In practice, these constraints manifest as a collapse of productivity in hybrid configurations: despite having one or two components in Nanoplasmid^TM^ format, any inclusion of a conventional plasmid reintroduces molecular ratio imbalances that reduce the triple-positive cell fraction to levels comparable to the all-conventional system, effectively nullifying the benefits of backbone minimization and optimised *Rep* regulation present in the Nanoplasmid^TM^. By contrast, the all-Nanoplasmid^TM^ system maintains matched backbone architecture and consistent molecule-number delivery across all three plasmids, preserving stoichiometry and enabling high-efficiency co-expression. This uniformity likely facilitates synchronous transcriptional and translational activity for *Rep* and helper genes [25,26], ensures proper capsid protein synthesis, and promotes efficient packaging of the transgene genome [27], collectively resulting in the observed ~10-fold enhancement in titre relative to conventional or hybrid systems. The findings are consistent with prior reports indicating that rAAV yield is strongly constrained by the lowest-performing component in triple-transfection systems, emphasising the critical importance of co-delivered plasmid balance for achieving maximal vector output [18,28]. Furthermore, these data underscore that the advantages of Nanoplasmid^TM^ design: compact backbones that reduce cellular stress and silencing, elimination of non-essential sequences that might interfere with nuclear processing, and controlled *Rep* expression via the truncated P5 promoter. This mechanistic insight might explain why single-component swaps fail to produce intermediate gains: any conventional plasmid reintroduces size-dependent dosing discrepancies, backbone heterogeneity, and potential differences in polyplex condensation or unpacking, disrupting synchronised expression and assembly. Collectively, these results demonstrate that the superior performance of Nanoplasmid^TM^ in rAAV9 production is emergent at the system level, reflecting a synergistic interplay between molecular architecture, intracellular delivery, and stoichiometric balance, rather than the independent contribution of any single plasmid. The findings also highlight the practical implication that optimising triple-transfection systems requires holistic consideration of all plasmid components; partial adoption of optimised constructs does not suffice to achieve high titres. In addition, the all-or-nothing outcome suggests that precise control of molecular ratios is paramount: any imbalance caused by size or backbone differences can negate the benefits of backbone minimisation, emphasising the importance of both molecular design and dosing strategy in complex multi-plasmid transfections. This insight aligns with prior observations in HEK293 suspension cultures that rAAV yields are highly sensitive to co-delivery efficiency and relative plasmid abundance [29], and that triple-positive fraction dictates the ceiling for production [18], with individual plasmid performance being necessary but insufficient on its own. Taken together, these experiments establish that Nanoplasmid^TM^-mediated improvements in rAAV9 production are contingent on concurrent implementation across all three plasmid components, validating a systems-level approach to plasmid engineering and highlighting the mechanistic basis for the dramatic, platform-wide increase in vector genome titres observed with all-Nanoplasmid^TM^ configurations. In addition, we have demonstrated manufacturability of an AAV product carrying a therapeutic human sequence, further validating Nanoplasmid^TM^ as suitable starting material for the production of clinically relevant gene therapy products. Taken together, these findings provide a robust framework for designing high-efficiency rAAV production systems: only by harmonising backbone architecture, plasmid size, and *Rep/Cap* regulatory features across helper, *Rep/Cap*, and transgene plasmids can maximal co-transfection and synchronised expression be achieved, resulting in the observed high-titre state. Further DoE optimisation evaluating other variables affecting polyplex formation, including DNA molar ratio and PEI:DNA ratio, will provide additional information on the optimal transfection and trans-complementation parameters.

Our findings demonstrate that Nanoplasmid^TM^ deployment can substantially enhance upstream productivity in transient AAV manufacturing processes. Nanoplasmid^TM^ substitution represents a structural modification of the transfection substrate rather than the vector itself. Several mechanisms may contribute to observed productivity gains: reduced plasmid size may improve nuclear uptake efficiency or reduce cytoplasmic degradation. In addition, backbone minimization may lower transcriptional interference while improving promoter balance between helper and *Rep/Cap* elements. Reduced bacterial sequence burden may also decrease innate immune activation or metabolic stress within transfected cells. Reduced plasmid size may improve nuclear entry efficiency, reduce transcriptional interference, and decrease metabolic burden during transfection. And finally, removal of antibiotic resistance genes may also attenuate cellular stress responses associated with CpG-rich bacterial sequences while reducing regulatory concerns associated with horizontal gene transfer risk [30]. Recent evidence [10] has shown that manufacturing-derived plasmid sequences can persist in vivo following AAV administration, highlighting the importance of minimising DNA impurities during vector production. In this context, Nanoplasmid^TM^ may offer a safety advantage because they contain highly reduced bacterial backbones and eliminate antibiotic resistance genes. Consequently, any inadvertent reverse-packaged DNA contaminants are likely to be fewer, smaller, and biologically less concerning than those derived from conventional plasmids. While Nanoplasmid^TM^ technology does not address broader issues such as vector genome rearrangements or integration events, they represent a practical strategy to reduce extraneous DNA burden.

Standard plasmids used in transient transfection are classified as critical starting materials under GMP regulations. Transitioning to Nanoplasmid^TM^ therefore requires full raw material qualification, including master cell bank establishment, sequence confirmation, endotoxin testing, and stability studies. From a regulatory perspective, because Nanoplasmid^TM^ substitution does not alter capsid sequence or vector genome design, the primary CMC considerations relate to process comparability rather than product identity. Key AAV critical quality attributes include vector genome integrity, capsid identity, potency, full:empty ratio, aggregation state, and residual impurities. As capsid sequence is unchanged, identity and antigenicity are expected to remain constant. However, increased productivity necessitates reassessment of full:empty ratios and residual plasmid DNA burden. If implemented during clinical development, Nanoplasmid^TM^ substitution would constitute a manufacturing process change requiring comparability assessment where further analytical confirmation is required.

GMP Plasmid DNA represents a significant contributor to AAV cost-of-goods. Enhanced yield per batch would reduce required plasmid mass and increase facility throughput efficiency. From a Quality-by-Design perspective, Nanoplasmid^TM^ substitution expands the upstream design space by increasing productivity margins and may improve robustness against minor process variability. By increasing yield without modifying capsid biology, Nanoplasmid^TM^ deployment offers a low-risk intensification strategy relative to capsid redesign or platform change [31]. Overall, Nanoplasmid^TM^ deployment represents a scalable upstream intensification strategy with favourable regulatory and CMC implications. Integration within manufacturing development programmes may contribute meaningfully to improving the economic sustainability and accessibility of AAV gene therapies.

## Data Availability

The original contributions presented in this study are included in the article. Further inquiries can be directed to the corresponding author(s).
